# Role of diffusion-weighted imaging in response prediction and evaluation after high dose rate brachytherapy in patients with colorectal liver metastases

**DOI:** 10.2478/raon-2024-0017

**Published:** 2024-02-21

**Authors:** Salma Karim, Ricarda Seidensticker, Max Seidensticker, Jens Ricke, Regina Schinner, Karla Treitl, Johannes Rübenthaler, Maria Ingenerf, Christine Schmid-Tannwald

**Affiliations:** Department of Radiology, University Hospital, LMU Munich, Germany; ENETS Centre of Excellence, Interdisciplinary Center of Neuroendocrine Tumours of the GastroEntero-Pancreatic System at the University Hospital of Munich (GEPNET KUM), University Hospital of Munich, Munich, Germany

**Keywords:** liver, HDR-brachytherapy, diffusion-weighted imaging, apparent diffusion coefficient, colorectal liver metastases

## Abstract

**Background:**

The aim of the study was to assess the role of diffusion-weighted imaging (DWI) to evaluate treatment response in patients with liver metastases of colorectal cancer.

**Patients and methods:**

In this retrospective, observational cohort study, we included 19 patients with 18 responding metastases (R-Mets; follow-up at least one year) and 11 non-responding metastases (NR-Mets; local tumor recurrence within one year) who were treated with high-dose-rate brachytherapy (HDR-BT) and underwent pre- and post-interventional MRI. DWI (qualitatively, mean apparent diffusion coefficient [ADCmean], ADCmin, intraindividual change of ADCmean and ADCmin) were evaluated and compared between pre-interventional MRI, first follow-up after 3 months and second follow-up at the time of the local tumor recurrence (in NR-Mets, mean: 284 ± 122 d) or after 12 months (in R-Mets, mean: 387+/−64 d). Sensitivity, specificity, positive predictive values (PPVs), and negative predictive values (NPVs) for detection of local tumor recurrence were calculated on second follow up, evaluating (1) DWI images only, and (2) DWI with Gd-enhanced T1-weighted images on hepatobiliary phase (contrast-enhanced [CE] T1-weight [T1w] hepatobiliary phase [hb])

**Results:**

ADCmean significantly increased 3 months after HDR-BT in both groups (R-Mets: 1.48 ± 0.44 and NR-Mets: 1.49 ± 0.19 x 10^−3^ mm^2^;/s, p < 0.0001 and p = 0.01), however, intraindividual change of ADCmean (175% *vs*.127%, p = 0.03) and ADCmin values (0.44 ± 0.24 to 0.82 ± 0.58 x 10^−3^ mm^2^/s) significantly increased only in R-Mets (p < 0.0001 and p < 0.001). ADCmin was significant higher in R-Mets compared to NR-Mets on first follow-up (p = 0.04). Sensitivity (1 *vs*. 0.72), specificity (0.94 *vs*. 0.72), PPV (0.91 *vs*. 0.61) and NPV (1 *vs*. 0.81) could be improved by combining DWI with CE T1w hb compared to DWI only.

**Conclusions:**

DW-MRI seems to be helpful in the qualitative and quantitative evaluation of treatment response after HDR-BT of colorectal metastases in the liver.

## Introduction

Image-guided interstitial high-dose-rate brachytherapy (HDR-BT) supported by CT or MR fluoroscopic-guided catheter implantation and dose calculation is a relatively new percutaneous ablation technique. It has shown promising results with consideration to safety, local tumor control, efficiency and overall survival (OS) in patients with unresectable liver metastases.^1,2,3,4^ HDR-BT can be performed repeatedly as therapy for recurrent liver metastases while maintaining liver function as high irradiation doses with steep dose gradients are being precisely applied to tumor tissue assuring the sparing of surrounding liver parenchyma.^5^

High-dose-rate brachytherapy (HDR-BT)^6^ of unresectable liver metastases leads to post-radiogenic changes such as post-radiogenic margins and vascularization, resulting in limited ability to assess morphological images^6,7^ similar to other loco-regional treatment (LRT) methods like radiofrequency ablation (RFA)^8,9^ or selective internal radiotherapy (SIRT).^10,11^

Currently, the modified response evaluation criteria in solid tumors (mRECIST) is used to evaluate treatment response of Hepatocellular carcinoma (HCC) after loco-regional treatment (LRT) strategies, based on tumor size and contrast agent enhancement.^12,13^ However, studies have shown that these criteria might be limited because post-treatment contrast enhancement are not exclusive characteristics of viable tumor and may also be seen in benign tissue as a result of inflammation or due to post-radiogenic changes. Thus, mRECIST may underestimate treatment response.^14,15,16^ However, tumor response evaluated by the RECIST 1.1 is a morphologically-based by assessing the change in the size of the tumor and do not take into account information about the intra-lesional features.^17^ On the other hand side colorectal liver metastases demonstrate peripheral rim enhancement on the arterial phase and appear hypointense in the portal venous phase with delayed phase of enhancement.^18^ If a tumor has residual rim enhancement on the post-treatment contrast-enhanced CT (CECT), it may have viable tumor components, as confirmed by pathological correlation.^19^

Diffusion-weighted imaging (DWI) reflects motion of free water molecules and allows qualitative and quantitative (on apparent diffusion coefficient [ADC] map) evaluation of changes in tissue cellularity.^9,20,21^ Therefore, it seems promising as a complement for evaluation of treatment response. Previous studies have already demonstrated the ability of DWI to assess tumor response in the liver after RFA as well as after SIRT to monitor different anticancer therapies.^10,22,23,24,25,26^ In addition, there are also studies showing that DWI may be an additional tool for predicting tumor response in patients with colorectal cancer liver metastases.^24,27,28^

However, there is limited data reflecting the role of diffusion-weighted imaging in evaluation of tumor response in patients with liver metastases treated with HDR-BT. Wybranski *et al*. showed that DWI is an important parameter for early prediction of treatment response after HDR-BT in patients with colorectal liver metastases.^5^ However, the study had an observation interval of only three months after therapy, and did not differentiate between responding and non-responding lesions.^5^

Therefore, the purpose of this study was to assess the role of diffusion-weighted imaging in response prediction and evaluation after HDR-BT in patients with colorectal liver metastases over short and long-term intervals.

## Patients and methods

### Patients

This retrospective observational cohort study was approved by the local research ethics committee and the need for written informed patient consent was waived. The reporting of this study conforms to the Strengthening the Reporting of Observational Studies in Epidemiology STROBE guidelines.^29^

Consecutively selected patients with liver metastases of colorectal cancer who were treated by HDR-BT at our department between August 2017 and December 2018 and who underwent MRI with DWI before, three months after HDR-BT and a second follow-up MRI at time of local tumor recurrence in nonresponding metastases (NR-Mets) and 12 months after therapy in responding metastases (R-Mets) were evaluated. Exclusion criteria were severe motion artefacts, lesion size less than 1cm, an incomplete MRI protocol, locoregional ablative therapy of treated metastases before and after HDR-BT.

#### CT-guided interstitial HDR-BT

Patient selection for treatment with CT-guided HDR-BT was based on a consensus decision in an interdisciplinary tumor conference. If multiple lesions were treated by brachytherapy in a patient, all treated lesions were included in the study. The procedure was performed in one single session as described before.^6,30^ After analgesia and sedation, CT-guided brachytherapy catheters were positioned inside the tumor volume, followed by a planning CT scan. The HDR-BT in after-loading technique was then performed using a ^192^Ir source. After irradiation, the catheters were removed while Gelfoam was administered to seal the puncture tract.^6,30^ The applied dose was at least 15 Gy surrounding the tumor.

#### MR imaging

Standardized pretreatment and posttreatment liver MRI were performed on a 1.5 T MR system (Magnetom Avanto, Magnetom Aera Siemens Healthcare, Erlangen, Germany or Ingenia, Ingenia S, Philips Healthcare, Best, Netherlands). Liver MRI included unenhanced T1w gradient-echo (GRE) (2D Flash) sequences in- and out-of-phase, a single shot T2w sequence (HASTE), T1w 3D GRE sequences with fat suppression (VIBE) before and 20, 50, and 120 seconds (depending on circulation time) after intravenous contrast injection (Gd-EOBDTPA; Primovist, Eovist, Bayer Schering Pharma, Germany; 25 μmol/kg body weight), a multishot T2w turbo spin echo sequence with fat saturation, diffusion-weighted sequences with b-values of 50, 400 and 800 s/mm^2^ and, after a delay of 15 minutes, an additional T1w GRE sequence with fat saturation (2D FLASH) and a fat suppressed T1w VIBE 3D GRE sequence identical to those performed earlier. Parallel imaging with an acceleration factor of 2 was utilized for all sequences. ADC maps were automatically computed from acquired DWI-MR images including all b-values.

### Image analysis

#### Standard of reference

Diagnosis of the primary tumor was established by histopathology. The evaluation of treatment response was lesion-based and based on mRECIST criteria on longterm follow-up imaging in consensus. Treatment evaluation was based on mRECIST but also included enhancement not only in the arterial but also in the portalvenous phase with corresponding hypointensity in the hepatobiliary phase:

Complete response (CR): Disappearance of any intratumoral enhancement
Partial resoinse (PR): (a) A decrease of vascularization of at least 30% or (b) a decrease of vascularization of at least 30% without washout or (c) decreasing defect/size (at least 30%) in the hepatobiliary phase
Progressive disease (PD): (a) over time increasing size and enhancement (at least 20%) or (b) new nodular enhancement with corresponding hypointensity
Stable disease (SD): Treated lesions were in between the three categories mentioned above.

R-Mets were defined as lesions (a) without vascularization in the sense of a disappearance of any intratumoral enhancement (CR) or (b) a decrease of vascularization of at least 30% without washout or (c) decreasing defect/size (at least 30%) in the hepatobiliary phase (PR) 12 months after therapy. Whereas NR-Mets were defined as treated lesions with (a) persisting (SD) and over time increasing (PD) size and enhancement (at least 20%) or (b) new nodular enhancement with corresponding hypointensity (PD) in the hepatobiliary phase.

#### Quantitative and qualitative image analysis

Image analysis was performed in consensus by two radiologists. The review was conducted in three separate sessions by two radiologists in consensus: (1) preinterventional MRI and (2) 1^st^ postinterventional MRI and (3) 2^nd^ post-interventional MRI with 2-week interval between the review sessions.

##### Location and size measurements

The location of each metastasis was recorded, and size measurements were performed on T1-weighted postcontrast imaging in the hepatobiliary phase on the slice with the largest tumor extent, excluding post-radiogenic changes, in consensus with all other acquired sequences.

##### Evaluation of metastases and normal liver on DWI

In each session, DWI was evaluated whether (1) metastases exhibited visually restricted diffusion or (2) showed no diffusion restriction (invisible on high b-value images or T2 shine through).

Mean ADC values of tumor-free hepatic parenchyma were measured on pre- and post-interventional DWI-MR images by placing circular ROIs, as large as possible, in areas of normal liver parenchyma.

For ADC measurements of the metastases circular regions-of-interest (ROI) were manually drawn on the slice with the largest tumor extent on diffusion-weighted images while excluding neighboring structures or regions close to the rim of the lesion to avoid partial volume effects. Then, ROIs were transferred to the same slice of the ADC map to calculate intralesional ADC values including minimal (ADCmin), and mean (ADCmean) ADC values (below noted as 10^−3^ mm^2^/s).

##### Evaluation of DWI for local tumor recurrence assessment

Furthermore, in two additional separate sessions, a third reviewer recorded the presence of local tumor recurrence on second follow up evaluating (1) DWI images only, (2) DWI with Gd-enhanced T1-weighted images on hepatobiliary phase (contrast-enhanced [CE] T1-weight [T1w] hepatobiliary phase [hb]) with a four points confidence scale: 1 = no local tumor recurrence, 2 = probably no local tumor recurrence, 3 = probably local tumor recurrence, 4 = definite local tumor recurrence.

On DWI, local tumor recurrence was defined as new or increasing nodular diffusion restriction over time. On CE T1w hb, local tumor recurrence was recorded if hypointense, treated metastasis either increased in size or new hypointense lesions appeared directly adjacent to the boarder of treated lesion.

### Statistical analysis

Statistical analysis was performed with IBM SPSS Statistics 22 (IBM Corporation) and SAS (version 9.4) for Windows (SAS Institute, Inc.).

All ADC values and size measurements by both readers were averaged for further statistical analysis. Statistical significance level was set at p ≤ 0.05. For normally distributed data, such as mean and min ADC of target lesions, paired t-tests were used for comparisons between study visits (before *vs*. after HDR-BT) and two-sample t-tests were used for comparisons between response groups (intraindividual changes in responders *vs*. non-responders). For non-normally distributed continuous data, such as lesion size, Mann-Whitney U test and Wilcoxon test were used instead of two-sample t-test and paired t-test. For categorical data, such as diffusion restriction McNemar’s test was used for comparisons between study visits, and Fisher’s exact test or Chi-square test was used for comparisons between response groups. Sensitivity, specificity, positive predictive values (PPVs), negative predictive values (NPVs) for detection of local tumor recurrence were calculated by means of cross tabulation. Significance levels of sensitivity and specificity of each review session were calculated using a McNemar Test. The Wilcoxon signed rank test for nonparametric paired samples was used for comparison of multiple confidence scores.

## Results

### Patients, MR interval, tumor location and size

**FIGURE 1.**
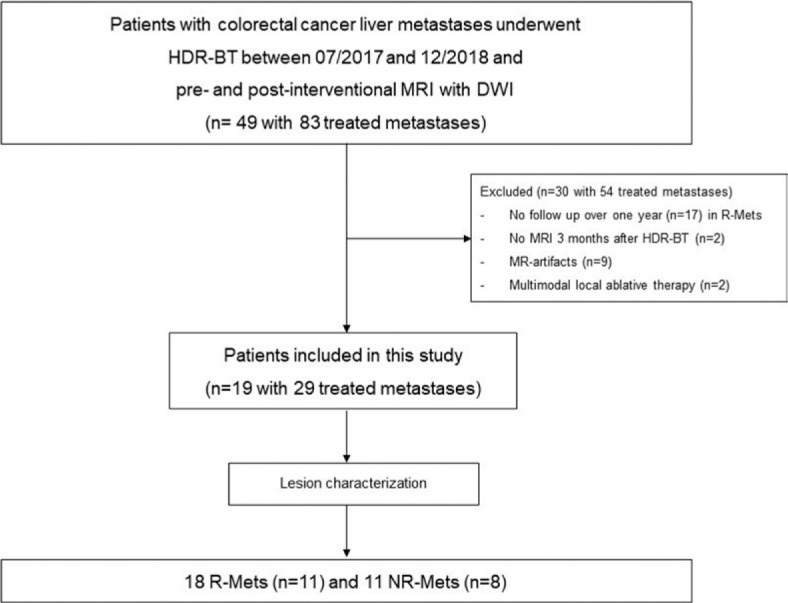
Flow diagram for this study DWI = diffusion-weighted imaging; HDR-BT = high-dose-rate brachytherapy; NR-Mets = non-responding metastases; R-Mets = responding metastases; HDR-BT = high-dose-rate brachytherapy

The final study population consisted of 19 patients (6 males, 13 females; mean age: 70 years, SD: 10.7) with a total of 29 treated liver metastases (Figure 1). According to reference standard, 18 metastases were rated as R-Mets in 11 patients and 11 metastases as NR-Mets in 8 patients. 11 patients were responders: 6 patients each had one responding lesion, 3 patients each had two responding lesions and 2 patients each had 3 responding metastases after treatment with brachytherapy. 8 patients were non-responders: 5 patients each had one NR-Mets, 3 patients each had two NR-Mets. There were no patients with both, responding and non-responding lesions.

Baseline imaging in R-Mets was performed 11 days (± 17 days) (MRI) before therapy, 1^st^ and 2^nd^ follow-up imaging were acquired 93 d (± 22 days) and 378 d (± 64 days) after HDR-BT, respectively.

Baseline imaging in NR-Mets was performed 21 days (± 17 days) before therapy, and 1^st^ and 2^nd^ follow-up imaging were acquired 96 d (± 36 days) and 284 d (± 122 days) (= at time of local recurrence) after HDR-BT, respectively.

11 lesions were located in the right lobe, and 18 lesions were located in the left.

The mean size of R-Mets was 2.2 ± 1.2 cm on preinterventional MRI and decreased significantly to 1.7 ± 0.9 cm on the first postinterventional images (p = 0.004) and showed another significant decrease (mean size 1.0 ± 0.4 cm) on the second postinterventional MRI (p = 0.0002). The mean size of NR-Mets also significantly decreased between pre-interventional MR images and the first postinterventional images (4.1 ± 2.2 cm and 3.3 ± 2.0 cm, respectively). However, on second follow-up, there was again a significant increase in size (mean size: 4.1 ± 2.3 cm, p = 0.02) (Table 1).

**TABLE 1.** Quantitative and qualitative results on baseline, 1. follow up and 2. follow up after local therapy of colorectal liver metastases with brachytherapy

**Target lesions**	**Responding metastases**			**Non-responding metastases**		
	**Baseline**	**1. follow-up**	**2. follow-up**	**Baseline**	**1. follow-up**	**2. follow-up**
**Size (cm)**	2.2 +/− 1.2	1.7 +/− 0.9	1.0 +/−0.4	4.1 +/− 2.2	3.3 +/− 2.0	4.1 +/− 2.3
**ADCmean**	0.84 +/− 0.34	1.44 +/− 0.19	1.48+/− 0.44	1.21 +/− 0.34	1.49 +/− 0.35	1.28 +/− 0.32
**ADCmin**	0.44 +/− 0.24	0.82 +/− 0.25	0.9 +/− 0.38	0.44 +/− 0.23	0.54 +/− 0.41	0.4 +/− 0.32
**Visually diffusion restriction**	11/18 (61.11%)	2/18 (11.11%)	0/18 (0%)	8/11 (72.38%)	4/11 (36.36%)	8/11 (72.38%)
**Intraindividual increase in**	**between baseline and 1. follow-up**	**between baseline and 2. follow up**		**between baseline and 1. follow-up**	**between baseline and 2. follow up**	
**ADCmean (%)**	175	187		127	106	
**ADCmin (%)**	208	281		146	115	

The size of R-Mets was significantly smaller than the size of NR-Mets on the pre-, 1^st^ post- and 2^nd^ postinterventional MRI (p = 0.007. 0.001 and p < 0.001, respectively).

#### ADC measurements

##### ADC of normal liver

Mean ADCmean of normal liver parenchyma for patients with R-Mets was 0.93 ± 0.11x 10^−3^ mm^2^/s on preinterventional images and 0.99 ± 0.16 x 10^−3^ mm^2^/s on 1^st^ postinterventional images and 0.88 ± 0.23 x 10^−3^ mm^2^/s on 2^nd^ follow up. Mean ADCmean of normal liver parenchyma for patients with NR-Mets was 0.92 ± 0.18 x 10^−3^ mm^2^/s on the preinterventional images and 0.84 ± 0.24 x 10^−3^ mm^2^/s on the 1^st^ post-interventional images and 0.96 ± 0.24x 10^−3^ mm^2^/s on the 2^nd^ follow up. There were neither a statistically significant change in mean ADC values of non-tumorous liver parenchyma between baseline and follow-up MRIs (p > 0.05) nor between responders and non-responders.

##### ADCmean of metastases

ADCmean of R-Mets (Table 1) was 0.84 ± 0.34 x 10^−3^ mm^2^/s on preinterventional images and 1.44 ± 0.19 x 10^−3^ mm^2^/s on the 1^st^ postinterventional images and 1.48 ± 0.44 x 10^−3^ mm^2^/s on the 2^nd^ follow up. There was a significant increase between baseline and the 1^st^ follow-up examination and between baseline and the 2^nd^ follow-up (p < 0.0001)

ADCmean of NR-Mets (Table 1) also increased significantly between pre- and 1^st^ postinterventional MRI (ADCmean: 1.21 ± 0.34 x 10^−3^ mm^2^/s to 1.49 ± 0.35 x 10^−3^ mm^2^/s) (p = 0.01); however, there was a significant decrease of ADCmean between 1^st^ and 2^nd^ follow up (ADCmean on 2. follow up: 1.28 ± 0.32 x 10^−3^ mm^2^/s, p = 0.04).

**FIGURE 2.**
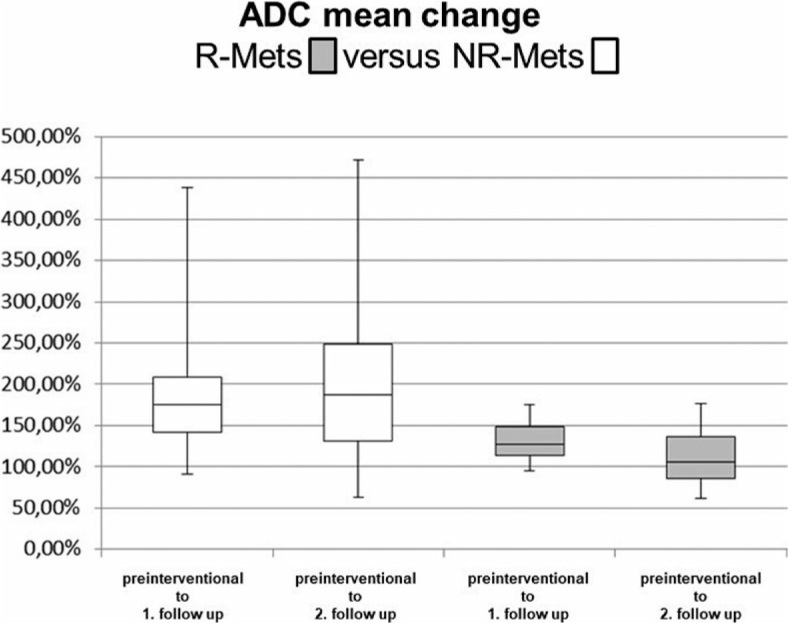
Mean apparent diffusion coefficient (ADCmean) change in responding metastases (R-Mets) and NR-Mets between preinterventional MRI and first and second follow-up, respectively ADC = apparent diffusion coefficient; NR-Mets = non-responding metastases, R-Mets = responding metastases

The intra-individual increase in ADCmean values of R-Mets after HD-BRT was 175% on first follow-up and 187% on second follow up compared to preinterventional MRI (Figure 2). The average increase in ADCmean values of R-Mets after HDBRT was 12% between first and second follow-up.

**FIGURE 3.**
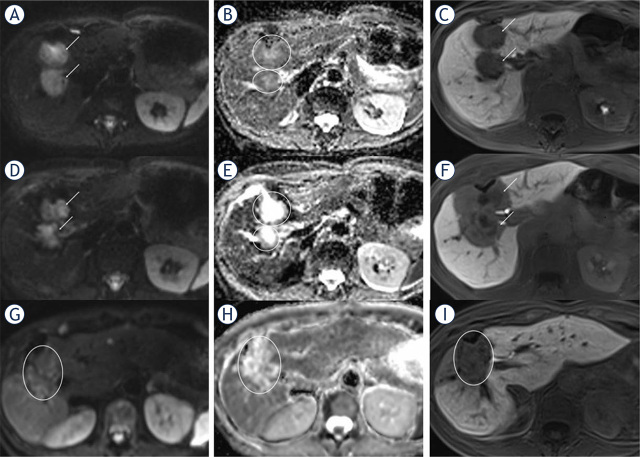
R-Met in a 62-year-old female. The pre-interventional diffusion-weighted imaging (DWI) shows two diffusion-restricted liver metastases with high signal on axial diffusion-weighted (DW)-MR image b = 800 s/mm^2^ **(A)** and low signal on apparent diffusion coefficient (ADC) map **(B)**. The pre-interventional ADCmean of the metastases were 0.83 and 0.86 x 10^−3^ mm^2^/s. In the hepatobiliary phase (**C**) both metastases showed a hypointense signal. After high-dose-rate brachytherapy (HDR-BT), the metastases demonstrated a hyperintense signal on the axial DW-MR image **(D)** and a hyperintense signal on the ADC map **(E)** indicating less restricted diffusion compared to the pre-interventional image. The ADCmean increased to 1.41 and 1.53 x 10^−3^ mm^2^/s. in the hepatobiliary phase **(F)**. The lesion showed central necrosis with a peripheral post-radiogenic hypointense rim. In the second follow-up the lesions showed no restricted diffusion **(G)** with a further increasing ADC **(H)** value of 2.09 and 2.07 x 10^−3^ mm^2^/s. There was a shrinkage in size of the metastases without a new hypointense defect in the hepatobiliary phase **(I)**.

**FIGURE 4.**
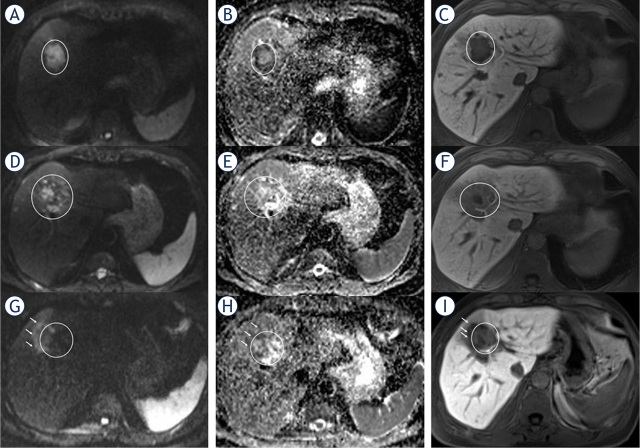
Non-responding metastases (NR-met) in a 56-year-old male. In preinterventional MRI, metastasis (circle) shows restricted diffusion **(A+B)** with an mean apparent diffusion coefficient (ADCmean) of 0.86 x 10^−3^ mm^2^/s and a hypointense pattern on the liver-specific phase **(C)**. Three months after high-dose-rate brachytherapy (HDR-BT), the metastasis showed visually partial restricted diffusion **(D+E)**, but, with an increasing ADCmean of 1.52 x 10^−3^ mm^2^/s and hypointensity in the hepatobiliary phase **(F)**. After 11 months, the lesion increased in size, shows a visually an increasing diffusion restriction **(K+L)** at the boarder (arrow) with a persistently ADCmean value of 1.53 x 10^−3^ mm^2^/s and a new defect in the hepatobiliary phase (arrow) **(I)** indicating local tumor recurrence.

The intra-individual increase in ADCmean values of NR-Mets after HD-BRT was 127% on first follow-up and 106% on second follow-up compared to preinterventional MRI (Figure 2). In contrast to R-Mets (Figure 3), there was a decrease of 21% in ADCmean values of NR-Mets (Figure 4) after HDBRT between first and second follow-up.

A cut-off value of a change of ADCmean less than 39% yielded a sensitivity of 0.82 (95% CI: 0.52–0.97) and a specificity of 0.72 (95% CI: 0.49–0.88).

Comparing the intra-individual change in ADCmean values between both groups, we found a significant difference between preinterventional ADCmean to ADCmean of the first and second follow up: (p = 0.03 and 0.01 retrospectively).

There was a significant difference of ADCmean between R-Mets and NR-Mets on preinterventional MRI (p = 0.008).

##### ADCmin of metastases

ADCmin of R-Mets (Table 1) increased significantly from 0.44 ± 0.24 x 10^−3^ mm^2^/s before treatment to 0.82 ± 0.25 x 10^−3^ mm^2^/s after treatment on the first follow up and to 0.9 ± 0.38 x 10^−3^ mm^2^/s on the second follow-up (p < 0.0001 and py0.001), but there was no significant increase of ADCmin between the first and second follow up (p = 0.49). In NR-Mets (Table 1) there was no significant change of ADCmin (from 0.44 ± 0.23 x 10^−3^ mm^2^/s to 0.54 ± 0.41 x 10^−3^ mm^2^/s to 0.40 ± 0.32 x 10^−3^ mm^2^/s) over time.

The intra-individual increase in ADCmin values of R-Mets after HD-BRT was 208% on first follow up and 281% on second follow up compared to preinterventional MRI.

The intra-individual increase in ADCmin values of NR-Mets after HD-BRT was 146% on the first follow up and 115% on the second follow up compared to preinterventional MRI.

There were no significant difference between preinterventional ADCmin to ADCmin of the second follow up: (p = 0.03) but not compared to first follow up (p = 0.1).

There was no significant differences of preinterventional ADCmin between R-Mets and NR-Mets, however ADCmin was significantly higher in R-Mets compared to NR-Mets on the first follow-up (p = 0.04).

#### Qualitative analysis of metastases

In R-Mets, there was a significant loss of diffusion restriction over time (pre- to first postinterventional MRI p = 0.012 and pre- to second postinterventional p = 0.001): On preinterventional MRI, 11/18 (61.11%) R-Mets were diffusion-restricted. On the first follow-up, only 2/18 (11.11%) showed diffusion restriction and on the second follow up, no responding metastasis showed restricted diffusion. In contrast, 8/11 (72.73%) NR-Mets showed restricted diffusion on preinterventional, 4/11 (36.36%) on the first follow up and then again 8/11 (72.73%) showed restricted diffusion on the second follow-up.

#### Detection of local tumor recurrence on DWI

There were 11 recurrent lesions in total on the second follow-up. On DWI only, 8 of 11 NR-Mets and 13 of 18 R-Mets were correctly detected. Combining DWI with the hepatobiliary phase 11 of 11 NR-Mets and 17 of 18 R-Mets were identified. It was not differentiated whether the lesions showed enhancement in the first follow-up and therefore showed a stable disease in the short-term interval (SD) or whether the lesions showed a completely new nodular enhancement with corresponding hypointensity on the hepatobiliary phase in the second follow-up (PD), since both types of lesions were classified as NR Mets.

The presence of local tumor recurrence on the second follow up evaluating DW-images only resulted in a sensitivity of 0.72, a specificity of 0.72, a positive predictive value (PPV) of 0.61 and a negative predictive value (NPV) of 0.81.

Combining DWI with Gd-enhanced T1-weighted images on hepatobiliary phase improved diagnostic performance: Sensitivity: 1, specificity 0.94, PPV: 0.91, NPV: 1.

## Discussion

Loco-regional treatment methods like SBRT or HDR-BT lead to post-radiation changes such as cell swelling, transudation of plasma components to the extravascular-extracellular and space of tumor but also cellular necrosis and changes in microvasculature.^5,6^ DWI reflects changes in tumor cellularity and cell membrane integrity but also vascular capillary perfusion.^20^ In the current literature quantitative and qualitative evaluation of DWI seems to be a promising tool in evaluating tumor response after loco-regional treatment; to the best of our knowledge there are no studies determining the role of DWI in patients with colorectal metastases treated with HDR-BT to stratify responding from non-responding metastases and therefore, determining the role of DWI for prediction of tumor response.

In the early follow-up, three months after treatment with HDR-BT, we found in both groups a significant increase of ADCmean. We suggest that high dose rate brachytherapy induces loss of cell membrane integrity, increased extracellular space and ultimately tumor cell lysis. However, regarding the intraindividual change of ADCmean, only responding lesions showed a significant increases in ADCmean which correlates with other studies in the literature.^25,31^ Furthermore ADCmin significantly increased in the responding lesions indicating necrosis induction in the tumor periphery beyond the already pre-interventional persisting central necrosis which is well known in colorectal metastases.^25,29,30,31,32^ It seems that DWI may be also used in long-term evaluation of tumor response. We found that over time (12months) a significant increase of ADCmean and ADCmin as well as loss of diffusion restriction could only be observed in responding metastases. In contrast, non-responding metastases even showed at time of local recurrence a decrease in ADCmean and ADCmin and consecutively increasing visual diffusion restriction compared to the first/early follow-up.

In the current literature there seems to be a disagreement regarding the role of pretreatment ADC values in predicting tumor response, depending on treatment techniques applied:

Cui *et al*. as well as Koh *et al*. demonstrated that pretreatment ADCmean values in non-responding lesions of patients with colorectal and gastric hepatic metastases treated by chemotherapy were significantly higher than those of nonresponding lesions.^22,24^ Before chemotherapy, the presence of necrosis (resulting in higher ADC values) may lead to less delivery of chemotherapeutic drugs to these less perfused regions.^24^

Similar results were found by Lahrsow *et al*.^28^: Responding colorectal liver metastases had significant lower ADC values than non-responding metastases before treatment with conventional lipiodol-based transarterial chemoembolization.

In contrast, Schmeel *et al*. showed that ADCmean in responding hepatic metastases of colorectal origin treated with 90Y-microsphere radioembolization were significantly higher than ADCmean of non-responding lesions.^27^ This might be attributed to the higher tumor grade and tumor aggressiveness associated with highly diffusivity restricted tumors.^27,33^

We found that preinterventional ADCmean was significantly lower in responding lesions compared to non-responding lesions. However, this result can only be seen as a marginal result and was not the central issue. In addition, as a limiting factor with regard to the value of our result, it must be noted that in our group the size of the metastases differed significantly between the responders and non-responders in the baseline examination.

In this study we could achieve good sensitivity and specificity in detection of local tumor recurrence; however, combining DWI with T1-weighted images in the hepatobiliary phase increased sensitivity, specificity and PPV and NPVv as well. Still, it must be noted, that there is a certain bias in the evaluation of combining DWI with T1-weighted images in the hepatobiliary phase, since T1-weighted images in the hepatobiliary phase was part of the gold standard that we have defined. However, at least one lesion was scored as a false positive combining DWI with T1-weighted images in the hepatobiliary phase, i.e. NR-Met, underlining that the contrast media behavior of the lesions should be part of the response assessment if possible. On the other hand, DWI alone achieves good but also worse results than the contrast-enhanced-based evaluation. Similar results were found by Liu *et al*. in detecting residual HCC after drug-eluting bead transarterial chemoembolization using DWI.34 Especially in very severe renal function impaired patients or in case of general avoidance of gadolinium exposure taking into account the frequent examinations over years in typical cases or in case of contraindication for contrast media administration DWI might be a valid alternative at imaging follow up after HDR-BT.

Our study has limitations due to its retrospective design, single center study and small sample size, which generally limits the conclusions to be drawn due to the lack of reproducibility. On the other hand, the evaluation was lesion-based with at least a total of 29 lesions were evaluated. Furthermore, the timing of imaging acquisition before and especially after treatment between the responder and non-responder group was not entirely similar. Moreover, mRECIST is limited by post-interventional changes that simulate a local recurrence. However, to overcome this limitation, we chose a long period after the intervention for the final evaluation. DWI is already routinely used to assess a treatment response of liver metastases and has been evaluated in many studies. Nevertheless, the use of DWI to assess a treatment response after brachytherapy in the liver has not yet been evaluated in short- and long-term studies; the post-radiogenic changes, especially after brachytherapy, can be delineated in a circular hypointensity in the hepatobiliary phase around the lesion for a relatively long time and thus the assessment of response is difficult, as a result providing the study basis.

In conclusion, our results indicate that DWIMRI may be a useful adjunct to morphologic MRI for detection of local tumor recurrence in patients with colorectal liver metastases treated with HDRBT.
